# Novel Secreted Protein of *Mycoplasma bovis* MbovP280 Induces Macrophage Apoptosis Through CRYAB

**DOI:** 10.3389/fimmu.2021.619362

**Published:** 2021-02-15

**Authors:** Gang Zhao, Xifang Zhu, Hui Zhang, Yingyu Chen, Elise Schieck, Changmin Hu, Huanchun Chen, Aizhen Guo

**Affiliations:** ^1^State Key Laboratory of Agricultural Microbiology, Huazhong Agricultural University, Wuhan, China; ^2^College of Veterinary Medicine, Huazhong Agricultural University, Wuhan, China; ^3^Key Laboratory of Development of Veterinary Diagnostic Products, Ministry of Agriculture, Huazhong Agricultural University, Wuhan, China; ^4^Hubei International Scientific and Technological Cooperation Base of Veterinary Epidemiology, Huazhong Agricultural University, Wuhan, China; ^5^Key Laboratory of Ruminant Bio-Products of Ministry of Agriculture and Rural Affairs, Huazhong Agriculture University, Wuhan, China; ^6^International Research Center for Animal Disease, Ministry of Science and Technology, Huazhong Agricultural University, Wuhan, China; ^7^International Livestock Research Institute, Nairobi, Kenya

**Keywords:** *Mycoplasma bovis*, MbovP280, secreted protein, apoptosis, CRYAB, macrophage

## Abstract

*Mycoplasma bovis* causes important diseases and great losses on feedlots and dairy farms. However, there are only a few measures to control *M. bovis*-related diseases. As in other mycoplasma species, this is predominantly because the virulence related factors of this pathogen are largely unknown. Therefore, in this study, we aimed to identify novel virulence-related factors among the secreted proteins of *M. bovis*. Using bioinformatic tools to analyze its secreted proteins, we preliminarily predicted 39 secreted lipoproteins, and then selected 11 of them for confirmation based on SignalP scores >0.6 or SceP scores >0.8 and conserved domains. These 11 genes were cloned after gene modification based on the codon bias of *Escherichia coli* and expressed. Mouse antiserum to each recombinant protein was developed. A western blotting assay with these antisera confirmed that MbovP280 and MbovP475 are strongly expressed and secreted proteins, but only MbovP280 significantly reduced the viability of bovine macrophages (BoMac). In further experiments, MbovP280 induced the apoptosis of BoMac treated with both live *M. bovis* and MbovP280 protein. The conserved coiled-coil domain of MbovP280 at amino acids 210–269 is essential for its induction of apoptosis. Further, immunoprecipitation, mass spectrometry, and coimmunoprecipitation assays identified the anti-apoptosis regulator αB-crystallin (CRYAB) as an MbovP280-binding ligand. An αβ-crystallin knockout cell line BoMac-cryab^−^, Mbov0280-knockout *M. bovis* strain T9.297, and its complemented *M. bovis* strain CT9.297 were constructed and the apoptosis of BoMac-cryab^−^ induced by these strains was compared. The results confirmed that CRYAB is critical for MbovP280 function as an apoptosis inducer in BoMac. In conclusion, in this study, we identified MbovP280 as a novel secreted protein of *M. bovis* that induces the apoptosis of BoMac via its coiled-coil domain and cellular ligand CRYAB. These findings extend our understanding of the virulence mechanism of mycoplasmal species.

## Introduction

*Mycoplasma bovis* is a member of the class *Mollicutes*, a group of the smallest self-replicating wall-less prokaryotes. It causes several important diseases, including pneumonia, mastitis, and arthritis, in cattle throughout the world (1–3). In addition, it usually co-infects cattle together with other pathogens, such as *Pasteurella multocida, Mannheimia haemolytica*, bovine viral diarrhea virus (BVDV), bovine respiratory syncytial virus (BRSV), *Bovine herpes virus 1* (BHV-1), etc to cause bovine respiratory disease complex (BRD) (4). Despite its minimal genome, *M. bovis* is a successful pathogen capable of developing both persistent infections and clinical diseases in cattle. As is well-known, *M. bovis*, like other mycoplasma species, lacks conventional toxins and its virulence mechanism is still poorly understood.

Although many previous studies have focused on the membrane and membrane-associated proteins of mycoplasmal species that are involved in virulence-related processes, such as adhesion (5), invasion (6, 7), and the inflammatory response (8), secreted proteins haven't yet attracted considerable attention until only recent years. However, secreted proteins often function as virulence-related factors or important antigens in pathogenic bacteria. Several studies have shown that the culture supernatant of *M. bovis* (9) induces the expression of several cytokines in different types of host cells and that live *M. bovis* behaves differently from the killed bacterium in inducing cytokine expression (10). Furthermore, several proteins of *Mycoplasma* species, including P80 of *M. hominis* (11), P102 of *M. hyopneumoniae* (12), Mpn491 of *M. pneumoniae* (13), CARDS toxin of *M. pneumoniae* (14), and a nuclease encoded by MBOV_RS02825 of *M. bovis* (15), have been shown to be secreted proteins. More recently, the secretomes and extracellular vesicles of several mycoplasmas species have been investigated with proteomic approaches, such as two-dimensional electrophoresis and liquid chromatography–tandem mass spectrometry (LC–MS/MS), isobaric tags for relative and absolute quantitation (iTRACK), and label-free proteomic analyses (16–18). However, the progress is relatively slow because it is usually difficult to confirm the secretomes and secreted proteins of mycoplasma species based on following reasons: (1) Mycoplasma species grow slowly and it is difficult to get sufficient proteins in short time; (2) Mycoplasma species growth requires rich medium supplemented with high concentrations of serum and yeast extract and it is difficult to exclude the contamination of abundant foreign proteins from the secretome in the culture supernatant; (3) There is no efficient genetic tools to manipulate gene expression of mycoplasma species by knock-out and knock-in to verify the predicted secreted proteins; (4) Most of the genes in mycoplasmal genomes are functionally unknown. One way dealing with this awkward situation tactfully is to combine the prediction of secreted proteins with bioinformatic tools, such as SignalP, SecretomeP, PSORT-B, and PrediSi (17–19) and identification of the secreted proteins with proteomic methods.

Therefore, in this study, we aimed to determine the novel secreted proteins of *M. bovis* and examine their association with *M. bovis* virulence. The secreted lipoproteins were first predicted with online softwares, and then the predicted secreted proteins were expressed and verified. Among the 11 predicted proteins, MbovP280 was identified as a secreted protein that induces apoptosis in a bovine macrophage cell line (BoMac) via the anti-apoptosis regulator αB-crystallin (CRYAB).

## Materials and Methods

### Ethics Statement

The protocols for the mouse experiments in this study were approved by the Experimental Animal Ethics Committee of Huazhong Agricultural University (Wuhan, China) (Permit number: HZAUMO-2018-027) and were performed in accordance with the Hubei Regulations for the Administration of Affairs Concerning Experimental Animals.

### Growth of Bacterial Strains and Cells

*Mycoplasma bovis* strain HB0801 (GenBank accession no. NC_018077.1) was isolated from the lesioned lung of an infected beef cattle from Yingcheng city in Hubei province, China and characterized by this laboratory (20). The strain was grown in pleuropneumonia-like organism (PPLO) medium (BD Company, Sparks, MD, USA), as previously described (20). *Mycoplasma bovis* mutants were grown in the same PPLO medium but supplemented with 100 μg/mL gentamycin or 10 μg/mL puromycin, depending upon the resistance genes the mutants carried. Recombinant *Escherichia coli* strains DH5α and BL21 (TransGen, Beijing, China) were grown in Luria–Bertani (LB) medium with antibiotics as necessary.

The BoMac cell line was kindly provided by Prof. Judith R. Stabel from the Johne's Disease Research Project at the United States Department of Agriculture in Ames, Iowa, and grown in RPIM 1640 medium (HyClone, UT, USA) supplemented with 10% heat-inactivated fetal bovine serum (FBS) (Gibco, Sydney, Australia) as described previously (21). The HEK293T cell line was purchased from the China Center for Type Culture Collection and cultured in high-glucose Dulbecco's modified Eagle's medium (HyClone) supplemented with 10% heat-inactivated FBS (Gibco).

### Prediction of Secreted Proteins Based on *M. bovis* HB0801 Genome

Classical secreted proteins were predicted with SignalP 4.1 (http://www.cbs.dtu.dk/services/SignalP/), while non-classical secreted proteins with SecretomeP 2.0 (http://www.cbs.dtu.dk/services/SecretomeP/) as described previously (19). The conserved domains in the predicted proteins were analyzed online with the National Center for Biotechnology Information (NCBI) Conserved Domain Database (http://www.ncbi.nlm.nih.gov/Structure/cdd/wrpsb.cgi). Proteins homologous to MbovP280 were identified with MolliGen 3.0 (http://services.cbib.u-bordeaux.fr/molligen/). The coiled-coil domain of MbovP280 was predicted with COILS (https://embnet.vital-it.ch/software/COILS_form.html). The homology models of CRYAB, caspase 3, and MbovP280 were generated with SWISS-MODEL (https://swissmodel.expasy.org/), and the interactions between CRYAB, caspase 3, and MbovP280 were analyzed with ClusPro 2.0 (https://cluspro.bu.edu/login.php).

### Gene Cloning and Expression of the Recombinant Proteins, and Polyclonal Antibody Production

The putative secreted lipoproteins with the highest prediction values and carrying conserved domains were selected for further analysis. First, the sequences of the selected genes were site-directedly edited by replacing the TGA codon with TGG to ensure that *M. bovis* tryptophan was correctly translated in *E. coli*. The sequences were synthesized by Beijing Tianyi Huiyuan Bioscience & Technology Inc. (Wuhan, China) and ligated into the pET-30a vector (Novagen, Darmstadt, Germany) (Supplementary Table 1). The modified genes were individually cloned into the pET-30a vector after digestion with restriction endonucleases *Bam*HI and *Xho*I. The MbovP280 mutant in which amino acids 210–269 (predicted to form a coiled-coil domain by the COILS software) were deleted was similarly cloned into pET-30a to generate pET-30a-Mbov_0280^Δ210−269^. *Escherichia coli* strain BL21 (TransGen, Beijing, China) was then transformed with each of the constructed plasmids individually, and the recombinant proteins were expressed after the cells were treated with isopropyl β-d-1-thiogalactopyranoside (IPTG) (0.8 mM). The proteins were purified with nickel affinity chromatography (GE Healthcare, NJ, USA), as described previously (7).

Mouse antisera against eight recombinant secreted proteins (rMbovP280, rMbovP290, rMbovP475, rMbovP458, rMbovP468, rMbovP537, rMbovP682, and rMbovP838) were produced in this study with a previously described method (7). Briefly, 4-week-old female BALB/c mice were purchased from China Hubei Provincial Center for Disease Control and Prevention (Wuhan, China) and raised in the Animal Facility of Huazhong Agriculture University. For each protein, five mice were immunized with 100 μg of each purified protein emulsified in an equal volume of Freund's complete adjuvant (Sigma, USA) for the priming immunization or with Freund's incomplete adjuvant for the subsequent immunization. Immunizations were performed by subcutaneous injection at an interval of 2 weeks. When the antiserum titers peaked, the mice were euthanized and bled. The antisera were collected and stored at −20°C for further use, and the preimmunization sera were stored for use as the negative controls.

Antibodies directed against the predicted secreted proteins rMbovP116, rMbovP275, and rMbovP739, which were previously developed by this laboratory (22), were also used in this study.

### Verification of the Secretion of Selected Proteins

The secretomes and whole-cell proteins of *M. bovis* were extracted to confirm the secreted nature of the predicted proteins with western blotting assays. The secretome of *M. bovis* was extracted with a previously described method (18). Briefly, *M. bovis* was cultured for 36 h to late log phase and harvested by centrifugation at 140,000 × g for 20 min. The bacterial debris in the supernatant was removed with a 0.22 μm filter. The filtered solution was then precipitated with 10% trichloroacetic acid (TCA), stored at 4°C overnight, and pelleted by centrifugation at 16,000 × g for 20 min at 4°C. The pellet was washed three times with cold acetone (−20°C) and resuspended in lysis buffer [8 M urea, 4% CHAPS, 2 M thiourea, 60 mM dithiothreitol, 2% amidosulfobetaine-14, 40 mM Tris-HCl (pH 8.8)]. The whole-cell proteins of *M. bovis* were prepared by sonicating the cells (200 W) on ice for 5 min. The protein concentrations of the secretome and whole-cell proteins were determined with the 2D Quant Kit (GE Healthcare, Sweden) and the BCA Protein Assay Kit (Cellchip Biotechnology Company, Beijing, China), respectively.

The western blotting assay was performed as follows. The secretome and whole-cell proteins were resolved with SDS-PAGE and then transferred onto polyvinylidene difluoride (PVDF) membranes (Millipore, Darmstadt, Germany). The mouse antisera directed against the 11 proteins and negative control sera were prediluted to 1:500 and separately overlaid onto the blotted PVDF strips. The proteins that specifically interacted with the antisera were visualized with WesternBright™ ECL (Advansta, CA, USA).

To detect the secretion process of MbovP280 and MbovP475 *in vitro*, the cultural supernatant of *M. bovis* HB0801 at 6, 12, 24, and 36 h was collected by centrifugation (15,400 g, 20 min, 4°C). Then, the supernatant (10 mL) was concentrated to 1 mL in an Amicon Ultra-4 Centrifugal Filter Unit (15 mL, 10 kDa) (Millipore). The equal volume of PPLO medium was concentrated as the negative control. The equal volume of supernatant (10 uL) from culture at each time point was resolved with SDS-PAGE and then transferred onto PVDF membranes (Millipore). The whole-cell proteins served as positive control and the membrane-associated protein NOX (7) of *M. bovis* was used as negative control. The bands were visualized by western blotting assay using the method described above.

### Observation of MbovP280 Binding With Confocal Microscopy

BoMac cells (1 ×10^5^) were propagated overnight on a microscope coverslip in each well of a 12-well-plate. To observe the binding of MbovP280 to BoMac, 0.5 μM MbovP280 was added to each well and incubated for 1 h at 37 °C. Phosphate-buffered saline (PBS) was used as the negative control. After the medium was removed, all the cells on the coverslips were washed three times with PBS, fixed with 4% paraformaldehyde in PBS for 10 min, and permeabilized with 0.5% Triton X-100 for 5 min at room temperature. All the cells were then blocked with 1% (w/v) bovine serum albumin (BSA) in PBS for 2 h. The cells were immunolabeled with mouse antiserum directed against rMbovP280 (1:500), and an Alexa-Fluor-488-conjugated goat anti-mouse IgG (H+L) secondary antibody (1:1,000) (Southern Biotech, MI, USA). Finally, the nuclei were counterstained with 4′,6-diamidino-2-phenylindole (DAPI) 5 mg/ml (Beyotime, Shanghai, China), and the polymerized form of actin was labeled with rhodamine phalloidin (100 nM) (Cytoskeleton, CO, USA). Finally, the slides were cover-slipped and observed with a confocal laser fluorescence microscope (Olympus FV1000 and IX81, Tokyo, Japan).

### Effect of MbovP280 on Cell Viability

The relative viability of BoMac and RAW264.7 cells after treatment with either recombinant MbovP280 (rMbovP280) or rMbovP475 was determined with a Cell Counting Kit-8 (CCK-8) (Dojindo Laboratories, Kumamoto, Japan). The cells were seeded at a density of 5,000 cells/well in 96-well-plates and incubated overnight at 37°C. They were then treated in triplicate with either rMbovP280 or rMboP475 at a concentration of 1 μM for 24 h. Cells treated with PBS were used as the negative control. CCK-8 (10 μl) was then added to each well, and the samples incubated for 2 h. The optical density at a wavelength of 450 nm (OD_450_) was measured and the relative cell viability was calculated as:

$$\begin{array}{l} \text{Relative cell viability }(\%)=({\text{OD}}_{\text{sample}}-{\text{OD}}_{\text{blank}})/({\text{OD}}_{\text{NC}}\\ \;-{\text{OD}}_{\text{blank}})\times 100\% \end{array}$$

The cells were then treated with either rMbovP280 or its mutant rMbovP280^Δ210−269^ at a concentration of 0.25, 0.5, or 1 μM for 24 h and the relative cell viability of BoMac was determined with the CCK-8 assay, as described above. Each treatment was carried out in five repeat and all experiments were performed independently three times.

### Screening for MbovP280-Binding Ligands With Immunoprecipitation (IP)–MS

An IP–MS method was used to screen for MbovP280-binding proteins. Briefly, BoMac cells were cultured, harvested, and lysed in RIPA buffer supplemented with cOmplete™ Protease Inhibitor Cocktail (Roche, Mannheim, Germany). The whole-cell lysates (400 μg) were incubated with 10 μg of either rMbovP280 or rMbovP280^Δ210−269^ for 1 h at 4°C. Mouse antiserum (5 μg) directed against MbovP280 was added to the lysates for 12 h at 4°C, and then 50 μl of Protein A/G Agarose Beads (Beyotime) were added and incubated for an additional 1 h. The immunoprecipitates were extensively washed with NP-40 buffer and eluted with SDS loading buffer by boiling them for 5 min. The cellular proteins co-precipitated with rMbovP280 or rMbovP280^Δ210−269^ were resolved with SDS-PAGE and stained with silver staining. The specific bands that bound only rMbovP280 were commercially sequenced and analyzed with MS by Applied Protein Technology (Shanghai, China).

### Verification of MbovP280-Binding Ligand CRYAB With Coimmunoprecipitation (Co-IP)

To confirm the interaction between MbovP280 and α-crystallin (CRYAB) in BoMac cells, the *CRYAB* gene (GenBank accession no. AF029793.2) was commercially synthesized at Beijing Tianyi Huiyuan Bioscience & Technology Inc. and cloned into pCAGGS-Flag, kindly provided by Prof. Xiao Shaobo (23). The Mbov_0280 gene (GenBank accession no. AFM51648.1) of *M. bovis* was synthesized and cloned into pCAGGS-HA, kindly provided by Prof. Xiao Shaobo at Huazhong Agricultural University, Wuhan, China (23). *Escherichia coli* strain DH5α (TransGen Biotech, Beijing, China) was transformed with the individual constructed plasmids, pCAGGS-Flag–CRYAB or pCAGGS-HA–Mbov_0280. The endotoxin-free plasmids were prepared with the E.Z.N.A.® Endo-Free Plasmid Mini Kit II (Omega Bio-tek, GA, USA) and then stored at −20°C until use. For the Co-IP assay, HEK-293T cells were cotransfected with both pCAGGS-Flag–CRYAB (8 μg) and pCAGGS-HA–Mbov_0280 (8 μg) using Lipofectamine 2000 (Invitrogen, Carlsbad, CA, USA) in a 10 cm dish. After 32 h, the cells were harvested and lysed in 1.5 ml of NP-40 buffer supplemented with cOmplete™ Protease Inhibitor Cocktail (Roche). The cell lysates (600 μl) were immunoprecipitated with 2 μg of commercial antibody directed against one of the recombinant tags [either Flag or hemagglutinin (HA)] for 12 h at 4°C. After the addition of Protein A/G Agarose Beads for 1 h, the immunoprecipitates were washed extensively with NP-40 buffer and eluted with SDS loading buffer by boiling for 5 min. A western blotting assay was then performed. The samples were resolved with SDS-PAGE and transferred to PVDF membranes (Millipore). The proteins were immunodetected with antibodies directed against either Flag (MBL, Nagoya, Japan) or HA (MBL, Nagoya, Japan). MbovP280 and CRYAB reacted with the antibodies directed against the corresponding tags on the PVDF membrane and were visualized with WesternBright™ ECL (Advansta).

### Construction of Strain Complementing the Mbov_0280 Mutant

The Mbov_0280-knockout mutant T9.297 was identified from a transposon-mediated *M. bovis* mutant library previously constructed in this laboratory (24). The mutated site was at nucleotide (nt) 418 of the Mbov_0280 coding sequence (CDS) or nt 323, 346 of the *M. bovis* HB0801 genome.

To construct a strain to complement the mutant T9.297, the sequence of the *M. agalactiae P40* promoter followed by the intact Mbov_0280 CDS was synthesized at Beijing Tianyi Huiyuan Bioscience & Technology Inc. and ligated into the pOH/P plasmid after digestion with restriction enzyme *Not*I, generating the recombinant plasmid pOH/P–Mbov_0280. T9.297 cells were transfected with pOH/P–Mbov_0280 to generate the complementing strain CT9.297, with a previously described method (25). Single colonies were selected with puromycin (10 μg/ml) in the medium and then confirmed with DNA sequencing. The T9.297 and CT9.297 strains were cultured in s medium containing 100 μg/ml gentamycin and 10 μg/ml puromycin, respectively, and their growth curves were determined with a colony counting method.

MbovP280 expression in mutant strain T9.297 and complementary strain CT9.297 was tested with western blotting assay. First, both strains were cultured in 20 ml of PPLO medium with the necessary antibiotics for 36 h and precipitated by centrifugation. The pellet of each strain was then suspended in 1 ml of PBS and lysed by sonication (200 W) on ice for 5 min. The proteins in the lysate were then separated with SDS-PAGE (10%) and transferred onto PVDF membrane (Millipore). The membrane was incubated with mouse antiserum (1:500) directed against rMbovP280 or rMbovP579 at room temperature for 1 h. After the membrane was washed, it was reacted with horseradish peroxidase (HRP)-conjugated goat anti-mouse IgG antibody (1:5,000; Southern Biotech) for 1 h at room temperature. The bands on the membrane were visualized with WesternBright™ ECL (Advansta).

### Construction of BoMac cryab^–^ Cell Line and Its Confirmation

CRISPR/cas9 gene editing was used to mutate the *CRYAB* gene in the BoMac cell line as described below. Single guide RNA (sgRNA) oligonucleotides to the *CRYAB* gene were designed with CCTop (https://cctop.cos.uni-heidelberg.de/) (Table 1). Ten microliter mixture of 100 μM *CRYAB* sgRNA oligonucleotides 1 (CRYABsgRNAoligo1) and 2 (CRYABsgRNAoligo2) (each 1 μl) (Table 1), NEB buffer 2 (1 μl), and ddH_2_O (7 μl) were prepared, and then annealed at 37°C for 30 min, heated at 95°C for 5 min, and cooled to 25°C. The lentiCRISPRv2 plasmid (Addgene plasmid #52961; http://n2t.net/addgene:52961; RRID: Addgene_52961) (26) was cut with *Bsm*BI and purified with the EasyPure® PCR Purification Kit (TransGen Biotech). Then 1 μl of the diluted (1:50) and annealed oligonucleotides was cloned into lentiCRISPRv2 with T4 ligase (New England Biolabs, Beijing, China) at 16°C for 12 h. Competent *E. coli* DH5α cells were transformed with the constructed plasmid plentiCRISPRv2-CRYABsgRNA and the construct was verified by sequencing with the primer U6-F (Table 1).

**Table 1:** Oligonucleotide primers used for PCR in this study.

**Names**	**Primer sequences (5^′^→3^′^)**
CRYABsgRNAoligo1	caccgTTCGGCCGCCCTCATTTCTG
CRYABsgRNAoligo2	aaacCAGAAATGAGGGCGGCCGAAc
U6-F	GAGGGCCTATTTCCCATGATTCC
CRYAB-C-F	AGCTCAGTGAGTACTGGGTAT
CRYAB-C-R	TGTAAGACAAAGGCCCCTTCT

The constructed plasmid plentiCRISPRv2-CRYABsgRNA and packaging plasmids pMD2.G (Addgene plasmids #12259; http://n2t.net/addgene:12259; RRID: Addgene_12259) and psPAX2 (Addgene plasmids #12260; http://n2t.net/addgene:12260; RRID: Addgene_12260) were amplified and purified with an Endo-free Plasmid Mini Kit II (Omega Bio-tek). The lentivirus was rescued as described previously (26) with some modification as follows. HEK293T cells were transfected with 12 μg of plentiCRISPRv2-CRYABsgRNA, 8 μg of psPAX2, and 4 μg of pMD2.G in 500 μl of jetPRIME® buffer (PolyPlus, Ilkirch, France) containing 48 μl of jetPRIME® reagent (PolyPlus). After 6 h, the medium was changed to DMEM containing 10% FBS and the cells were further cultured for 48 h. The lentivirus was harvested by centrifugation at 3,000 × g at 4°C for 10 min and then filtered through a 0.45 μm membrane (Millipore), concentrated 10-folds with Lenti-Pac™ Lentivirus Concentration Solution (GeneCopoeia, MD, USA), purified as described in the manual, and resuspended in 1 ml of PBS.

The BoMac cells with 50% confluency in each well of a 24-well-plate were infected with 100 μl of the purified lentivirus for 12 h. The medium was then changed to RPMI 1640 containing 10% FBS and 1% penicillin–streptomycin, and the cells were incubated for 36 h more. The media in the infected and uninfected wells were changed to fresh RPMI 1640, as described above, but with the addition of 2 μg/ml puromycin. After 3 days, the adherent cells were propagated in one well of a six-well-plate and cultured in RPMI 1640 (10% FBS, 1% penicillin–streptomycin) with 2 μg/ml puromycin for 3 days. The cells in the wells of the six-well-plate were then counted and 10-fold serially diluted in RPMI 1640 to a final concentration of 1 cell per 100 μl. The diluted cells (100 μl) were seeded to the wells of two 96-well-plates. The clonal cell lines were isolated from the 96-well-plates after 7 days, and expanded for 14 days.

The genome of the selected mutated cell line in each well was isolated with the TIANamp Genomic DNA Kit (Tiangen, Beijing, China). Q5 High-Fidelity 2 × Master Mix (NEB, Beijing, China) and the primers CRYAB-C-F and CRYAB-C-R (Table 1) were then used to amplify the sequences edited by CRISPR/cas9. The whole-cell proteins of each mutated cell line and those of the wild type (WT) were prepared in RIPA lysis buffer (Beyotime) and then resolved with SDS-PAGE, transferred onto PVDF membrane (Millipore), and incubated with a mouse monoclonal antibody directed against CRYAB (1:1,000) (Abcam, ON, Canada) at room temperature for 1 h. The membranes were then incubated with a HRP-conjugated goat anti-mouse IgG antibody (1:5,000) (Southern Biotech) for 1 h at room temperature. The bands on the membrane were visualized with Western Bright™ ECL (Advansta).

### Apoptosis of BoMac Specifically Induced by MbovP280

To examine the specific effect of rMbovP280 on the induction of BoMac apoptosis, the WT BoMac and mutant BoMac cryab^−^ cells were seeded at a density of 5 ×10^5^ cells per well in six-well-plates and incubated overnight at 37°C. They were then treated with rMbovP280 or its mutant rMbovP280^Δ210−269^ at three concentrations (0.25, 0.5, or 1 μM) for 24 h.

To observe the effect of MbovP280 expression in *M. bovis* strains on cell apoptosis, 5 ×10^5^ BoMac cells were seeded into each well of a six-well-plate and incubated overnight. The cells were then infected with WT *M. bovis* HB0801, the mutant T9.297, or complementary strain CT9.297, respectively, at an MOI of 1,000 for 24 h. The cells were harvested and stained with the Annexin V-FITC Apoptosis Detection Kit (Vazyme, Nanjing, China) according to the manufacturer's instructions (15). An Apoptosis Inducer Kit (Beyotime) was used as the positive control. Flow cytometry (BD Company, NJ, USA) was used to detect the apoptotic cells, and the data were analyzed with the FlowJo VX software. The experiments were performed independently three times.

To confirm the apoptosis determined by Annexin V-FITC Apoptosis Detection Kit, the levels of cleaved caspase-3 in BoMac cells infected with *M. bovis* or treated with rMbvoP280 and rMbovP280^Δ210−269^ as described above were detected by western blotting assay. The cells were washed with cold PBS, resuspended with RIPA buffer containing proteinase and phosphatase inhibitors, and lysed at 4°C for 1 h. The proteins in the lysates were then separated with SDS-PAGE (10%) and transferred onto PVDF membrane (Millipore). The membrane was incubated with cleaved caspase-3 (Asp175) antibody (Cell Signaling Technology, DANVERS, MA, USA) (1:1,000) at 4°C overnight after blocking with 5% skimmed milk. After the membrane was washed, it reacted with HRP-conjugated goat anti-mouse IgG antibody (1:5,000) (Southern Biotech) for 1 h at room temperature. The bands on the membrane were visualized with WesternBright™ ECL (Advansta).

### Statistical Analysis

Data were expressed as means ± standard error of mean (SEM). Student's *t*-test was used for single comparison and one-way ANOVA for multiple comparisons with the GraphPad Prism version 5 software (GraphPad Software, La Jolla, CA, USA).

## Results

### Prediction of Secreted Proteins Based on the Genome of *M. bovis* HB0801

A total of 39 secreted lipoproteins were predicted with a genome-wide analysis of *M. bovis* based on the SignalP-TM score and the SecP score (Supplementary Table 1). Among these lipoproteins, 19 were non-classical secreted proteins, four were classical secreted proteins, and 16 were non-classical/classical secreted proteins. Using the criteria of a SignalP score >0.6 or SceP score >0.8 and the prediction of conserved domains, 11 of the predicted secreted lipoproteins were selected for further investigation, including two classical, four non-classical, and five non-classical/classical secreted proteins, and seven of which contained conserved domains. For example, MbovP537 contains the aromatic cluster surface protein domain, which suggests its surface expression; MbovP458 contains the PAP1 superfamily domain, which may regulate the transcription of antioxidant genes in response to H_2_O_2_; and MbovP682 contains the PRK08581 domain, which may function as an N-acetylmuramoyl-l-alanine amidase (Table 2).

**Table 2:** Information on 11 predicted secreted proteins.

**Genes**	**Protein**	**SignalP Score**	**SecP Score**	**Domain**
Mbov_0739	Lipoprotein	<0.6	0.83	N
Mbov_0537	Lipoprotein	<0.6	0.81	Aromatic cluster surface protein
Mbov_0275	Lipoprotein	<0.6	0.90	N
Mbov_0475	Lipoprotein	<0.6	0.83	DUF285
Mbov_0280	Lipoprotein	0.60	<0.8	N
Mbov_0116	Lipoprotein	0.61	0.86	N
Mbov_0458	Lipoprotein	0.61	0.93	PAP1 super family
Mbov_0468	Lipoprotein	0.61	0.90	DUF31
Mbov_0290	Lipoprotein	0.61	<0.8	Leucine-rich repeat 5
Mbov_0838	Lipoprotein	0.69	0.91	DUF285
Mbov_0682	Lipoprotein	0.72	0.90	PRK08581

*SignalP score and SecP score were determined with SignalP 4.1 (http://www.cbs.dtu.dk/services/SignalP/) and SecretomeP 2.0 (http://www.cbs.dtu.dk/services/SecretomeP/), respectively. “N” indicates none predicted. “DUF” indicates domain of unknown function*.

### Confirmation of the Expression and Secretion of Selected Proteins

**Figure 1:**
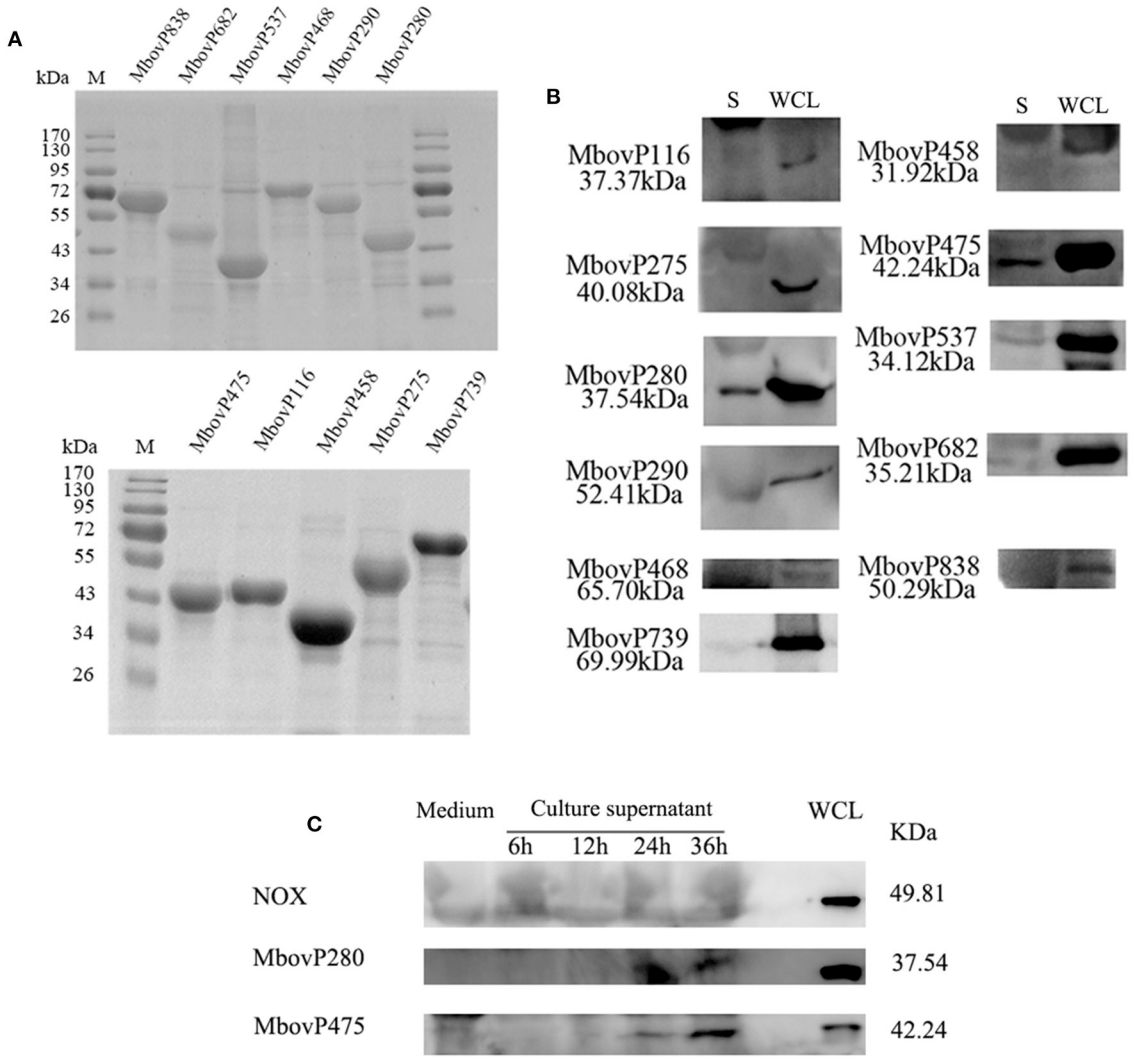
Secretion of MbovP280. **(A)** Purification of 11 predicted secreted proteins. The proteins were purified with nickel affinity chromatography and resolved with SDS-PAGE. **(B)** Confirmation of the predicted secreted proteins with western blotting assays. Secretome (S) and whole-cell lysate (WCL) of *M. bovis* were resolved with SDS-PAGE and transferred onto polyvinylidene difluoride membranes. Polyclonal antibodies directed against rMbovP280, rMbovP290, rMbovP475, rMbovP468, rMbovP838, rMbovP537, rMbovP458, rMbovP682, rMbovP116, rMbovP275, and rMbovP739 were used to detect the proteins in the secretome. **(C)** Visualization of the secreted MbvoP280 in culture supernatant. *M. bovis* HB0801 was cultured in PPLO medium. The culture supernatant was collected and concentrated at 6, 12, 24, and 36 h after incubation. The antiserum against rMbovP280 and rMbovP475 were used to detect the proteins in the supernatant, while *M. bovis* NOX known as the membrane-associated protein served as the negative control (NC).

The 11 selected genes of *M. bovis* were modified appropriately and cloned into *E. coli*. The recombinant proteins were shown to be correctly expressed, with the expected molecular masses, with SDS-PAGE (Figure 1A). The antiserum against each recombinant protein was used to detect the presence of each protein in the secretome and whole-cell proteins of *M. bovis* HB0801 with western blotting assays. The results indicated that all the proteins were present in large amounts in the whole-cell proteins, but only MbovP280 and MbovP475 were abundant in the secretome, whereas the other proteins were represented by only variously weak bands in the secretome (Figure 1B, Supplementary Figure 1). In order to detect the secretion process of MbovP280 and MbovP475 *in vitro*, the presence of MbovP280 and MbovP475 in the culture supernatant of *M. bovis* was kinetically examined, while the membrane-associated protein NOX served as the negative control. The results indicated the MbovP475 was detected in the supernatant after *M. bovis* was cultured for 24 h. But MbovP280 wasn't detected in the supernatant until *M. bovis* was cultured for 36 h. The membrane-associated protein NOX of *M. bovis* wasn't detected in the culture supernatant as expected (Figure 1C, Supplementary Figure 2).

### The rMbovP280 Reduces Cell Viability

**Figure 2:**
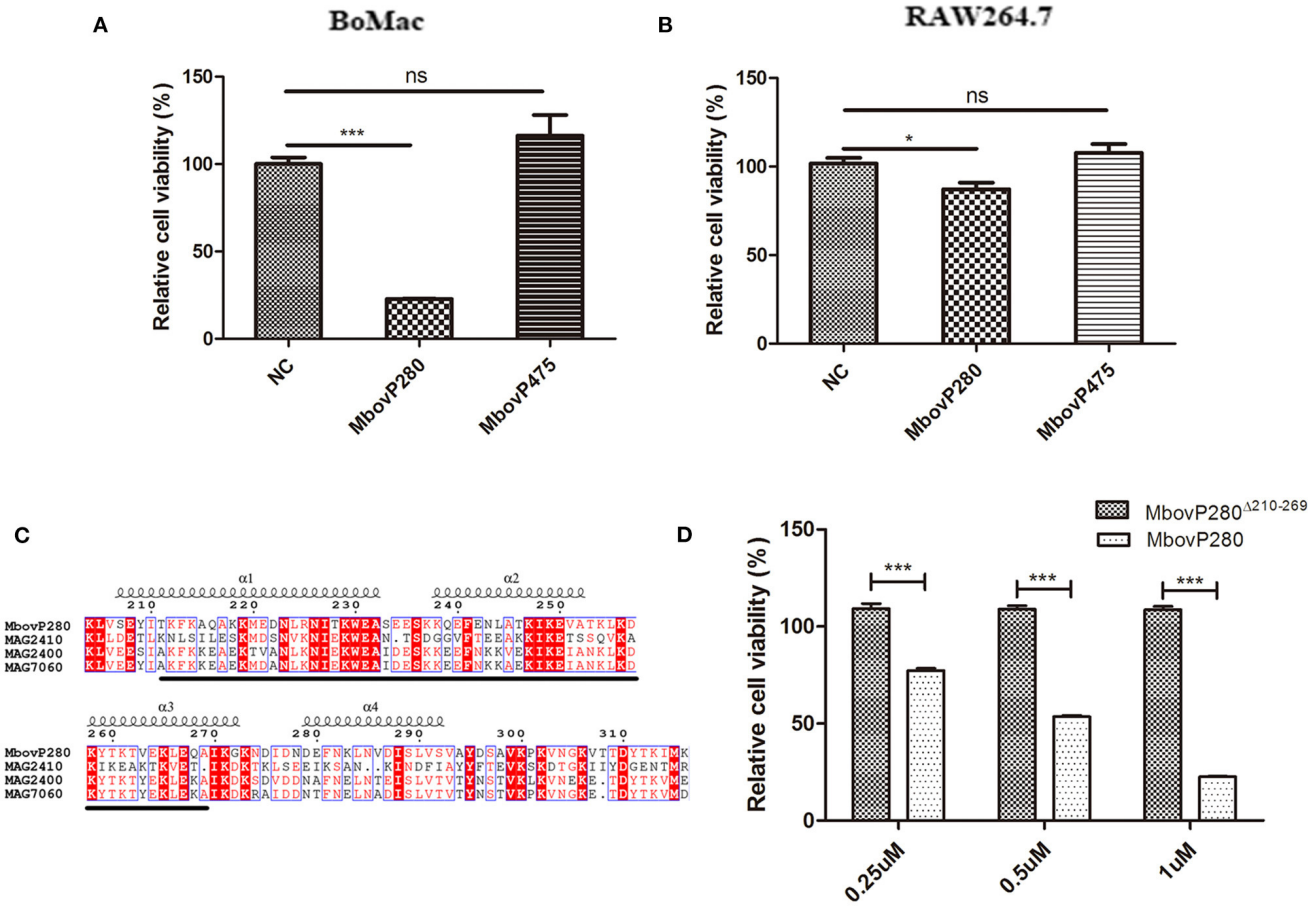
Viability change of macrophages stimulated with the recombinant proteins of *M.bovis*. **(A)** BoMac cells (5 ×10^3^ per well) or **(B)** RAW264.7 cells (1 ×10^4^) were treated with either 1 μM MbovP280 or 1 μM MbovP475 for 24 h, and then CCK-8 was added to each well to determine cell viability. Wells treated with PBS were used as the negative control (NC). **(C)** Proteins homologous to MbovP280 in *Mycoplasma agalactiae* were identified with MolliGen 3.0 (http://services.cbib.u-bordeaux.fr/molligen/). Amino acids 210–269 (black underline) were predicted to form a coiled-coil domain. A protein alignment was constructed with the ClustalW multiple sequence alignment programs (https://www.ebi.ac.uk/Tools/msa/clustalw2/) combined with ESPript 3.0 (http://espript.ibcp.fr/ESPript/ESPript/). **(D)** Relative cell viability of BoMac (5 ×10^3^ cells per well) treated with MbovP280 or MbovP280^Δ210−269^ at concentrations of 0.25, 0.5, or 1 μM for 24 h was tested with a CCK-8 assay. *p <0.05, ***p <0.001 indicate statistically significant differences; while ns indicates no difference.

The effects of rMbovP280 and rMboP475, at concentrations of 1 μM, on the viability of BoMac and RAW264.7 cells were determined with a CCK-8 kit after the cells were treated for 24 h. The viability of mock-treated cells as the negative control was taken as 100%. As a result, the rMbovP280 significantly reduced cell viability in a cell-type-dependent way. The rMbovP280 caused the viability a reduction of ~77.3% in BoMac cells (*p* <0.001), but a reduction of only about 12.9% in RAW264.7 cells (*p* <0.05). This is in agreement with our expectation that the macrophages of bovine origin is more sensitive to MbovP280 of *M. bovis*, the bovine pathogen, than the cells of mouse origin. However, rMbovP475 had no significant effect on the viability of either BoMac or RAW cells (Figures 2A,B).

A BLAST analysis with MolliGen 3.0 revealed that MbovP280 only shares some homology with genes in *M. agalactiae* strain PG2, including MAG2400 (GenBank accession no. CAL58938.1, similarity = 62%), MAG2410 (GenBank accession no. CAL58939.1, similarity = 36%), and MAG7060 (GenBank accession no. CAL59406.1, similarity = 31%). MbovP280 was also predicted to contain a conserved coiled-coil domain in the fragment defined by amino acids 210–269, which was predicted with COILS. This domain is potentially involved in protein–protein interactions (Figure 2C). To verify the role of the coiled-coil domain in reducing cell viability, the Mbov_0280 gene was mutated by deleting the coiled-coil domain, and the resultant recombinant protein MbovP280^Δ210−269^ was expressed. The relative viability of BoMac treated with different concentrations of rMbovP280 and rMbovP280^Δ210−269^ was determined in parallel, with a CCK-8 kit, and decreased significantly as the rMbovP280 concentration increased from 0.25 μM to 1 μM (*p* <0.001). However, treatment with rMbovP280^Δ210−269^ at any concentration (0.25, 0.50, or 1 μM) had no effect on BoMac viability (Figure 2D).

### Induction of BoMac Apoptosis by MbovP280 Is Associated With Expression of Cellular CRYAB

**Figure 3:**
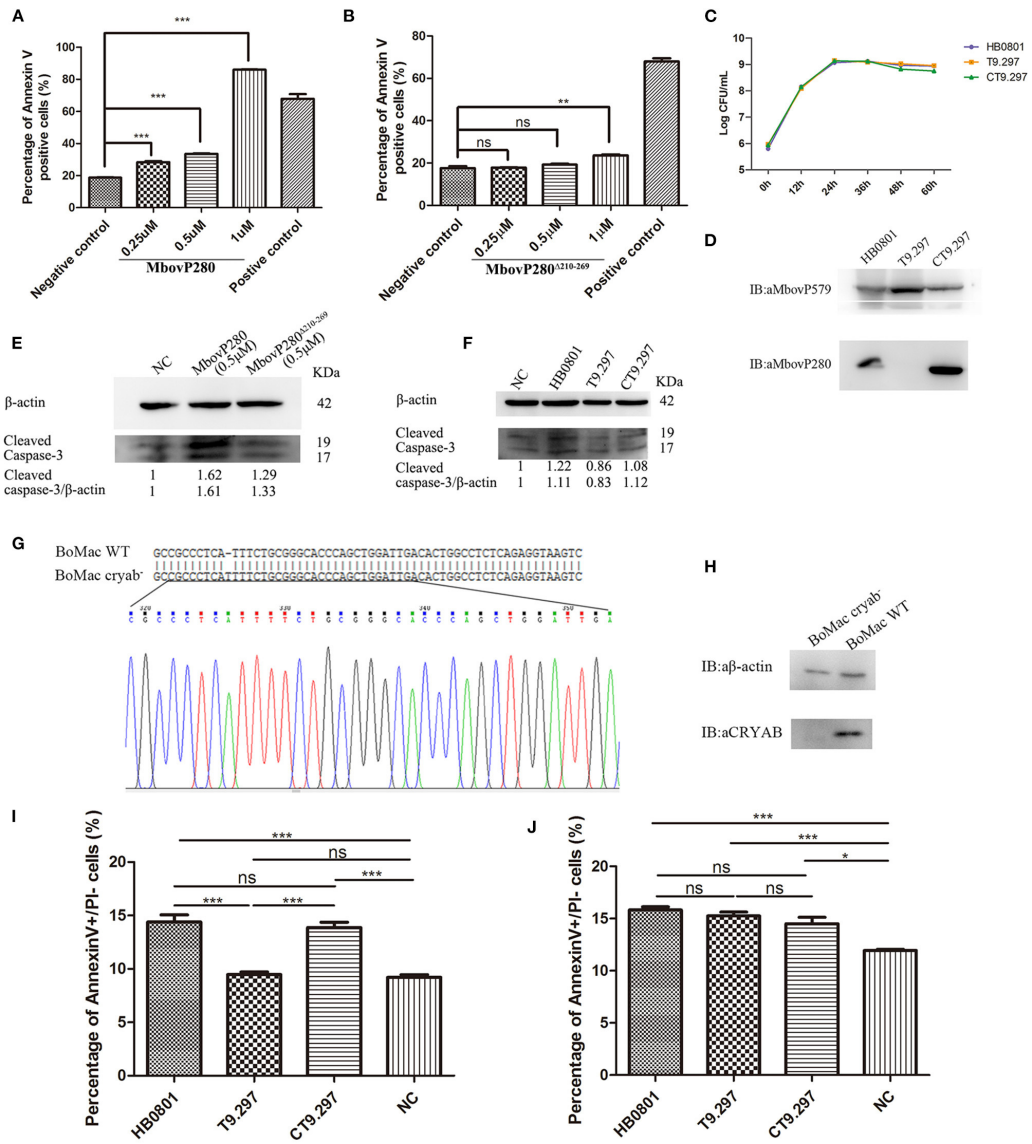
Apoptosis of BoMac cells induced by MbovP280 in mycoplasmas cells or as the recombinant protein. Cells (5 ×10^5^ per well) were treated with MbovP280 **(A)** or MbovP280^Δ210−269^ **(B)** at concentrations of 0.25, 0.5, or 1 μM for 24 h. The cells were then stained with annexin V and propidium iodide (PI) and detected with flow cytometry. **(C)** Growth curves of strains HB0801, the Mbov_0280 mutant T9.297, and its complement CT9.297. Growth of *M. bovis* at each time point was determined with a plating assay. **(D)** Visualization of MbovP280 expression in T9.297 and CT9.297 strains with a western blotting assay. Wild-type strain HB0801 was used as the positive control. **(E)** The cleaved caspase-3 of BoMac cells treated with rMbovP280. The cell lysates of BoMac treated with 0.5 μM rMbovP280 or rMbovP280^Δ210−269^ were resolved with SDS-PAGE, transferred onto membrane, and then immunodetected with an antibody directed against cleaved caspase-3. β-actin was used as the internal control. The ratio of the amount of cleaved caspase-3 to the amount of β-actin was calculated and normalized to the NC. **(F)** The cleaved caspase-3 of BoMac cells infected with *M. bovis*. The cell lysates of BoMac infected with HB0801, T9.297, or CT9.297 (MOI = 1,000) were resolved with SDS-PAGE, transferred onto PVDF membrane, and then immunodetected with the antibody directed against cleaved caspase-3. β-actin was used as the internal control. The ratio of the amount of cleaved caspase-3 to the amount of β-actin was calculated and normalized to the NC. **(G)** Single peak around the protospacer adjacent motif (PAM) in the sequence of BoMac cryab^−^ clonal cell line. BLAST analysis of sequences around the PAM sequence of WT BoMac (BoMac WT) and BoMac cryab^−^ cell lines. **(H)** Expression of CRYAB in BoMac WT and BoMac cryab^−^ cells. Whole-cell lysates of BoMac WT and BoMac cryab^−^ cells were resolved with SDS-PAGE, transferred onto membrane, and then immunodetected with the antibody directed against CRYAB. β-actin was used as the internal control. **(I)** Early apoptosis of BoMac induced by HB0801, T9.297, or CT9.297 was compared. BoMac (5 ×10^5^ cells per well) were treated with 5 ×10^8^ CFU of HB0801, T9.297, or CT9.297, or with PBS for 24 h. The cells only stained with annexin V are defined as undergoing early apoptosis. **(J)** Early apoptosis of BoMac-cryab^−^ cell line induced by *M. bovis*. BoMac (5 ×10^5^ cells per well) were infected with HB0801 (5 ×10^8^ CFU), T9.297 (5 ×10^8^ CFU), or CT9.297 (5 ×10^8^ CFU), or treated with PBS (NC) for 24 h. *p <0.05, **p <0.01, ***p <0.001 indicate statistically significant differences; ns indicates no difference.

To investigate the mechanism underlying the reduction in cell viability induced by MbovP280, the ability of rMbovP280 to reduce cell viability of BoMac was assayed with CCK-8. This assay demonstrated that rMbovP280 significantly reduced the cell viability of BoMac in a dose-dependent way (between 0.25 and 1.00 μM) (Figure 3A). In contrast, the mutant MbovP280^Δ210−269^ at 0.25 and 0.50 μM did not apparently reduce the cell viability of BoMac presenting the levels of cell viability similar to those in the negative control. However, 1.00 μM MbovP280^Δ210−269^ reduced cell viability, but the level was much lower than that in the rMbovP280-treated group (Figure 3B).

Further, the Mbov_0280 knockout mutant T9.297 and its complementary strain CT9.297 in which the fragment of the whole gene Mbov_0280 was inserted into the mutant T9.297, were constructed and confirmed with DNA sequencing. Although the growth curves of the three strains (HB0801, CT9.297, and T9.297) did not differ significantly (Figure 3C), the western blotting assay demonstrated that MbovP280 was expressed in both *M.bovis* HB0801 and CT9.297 strains, but not in the mutant T9.297 strain (Figure 3D).

In order to further verify the apoptosis of BoMac specifically induced by MbovP280, the levels of cleaved caspase-3 in BoMac cells either infected with *M. bovis* strains or treated with rMbovP280 or its mutant were detected by western blot assays. As a result, the rMbovP280 increased the amount of cleaved caspase-3, while the rMbovP280^Δ210−269^ did not (Figure 3E, Supplementary Figure 3). For the infection with *M. bovis* strains, the wild type strain HB0801 and complementary strain CT9.297 increased the levels of cleaved caspase-3 in BoMac, however the Mbov_0280 knock-out mutant T9.297 didn't (Figure 3F, Supplementary Figure 3).

The *CRYAB* gene of BoMac was knocked out with the CRISPR-Cas9 lentiviral system. Then the genome of the *CRYAB*-knockout cell line (BoMac-cryab^−^) was isolated and used as the template to amplify the edited sequence. The sequencing results indicated that the *CRYAB* gene was correctly mutated by the insertion of an additional T at nt 159 in the CDS of the *CRYAB* gene (Figure 3G). The deficiency of CRYAB expression in the clonal cell line (BoMac-cryab^−^) was confirmed with a western blotting assay (Figure 3H).

Further, the flow cytometry was used to test the apoptosis of BoMac-cryab^−^ cell line and its WT BoMac induced by the three strains of *M. bovis*: HB0801, the Mbov_0280 knock-out mutant T9.297, and its complementary strain CT9.297 after stained with annexin V and PI. As a result, the proportions of the cells at the early stage of apoptosis stained only by annexin V were apparently associated with MbovP280 stimulation. In WT BoMac, the apoptosis levels induced by HB0801, T9.297, CT9.297, and PBS were 14.40, 9.48, 13.87, and 9.21%, respectively. There is no difference in the apoptosis levels between HB0801/CT9.297; and T9.297/NC (PBS) groups (*p* > 0.05); However, there is a significant difference between HB0801/T9.297, CT9.297/T9.297, HB0801/NC, and CT9.297/NC (*p* <0.001) (Figure 3I). In contrast, in BoMac-cryab^−^ cells, the levels of apoptosis induced by HB0801 (15.83%), T9.297 (15.27%), and CT9.297 (14.5%) did not change significantly (*p* > 0.05), although all three strains indeed generated some degree of cellular apoptosis compared to NC (*p*<*0.05*) suggesting there is some other mechanism in *M. bovis* to induce BoMac apoptosis independent of MbovP280/CRYAB (Figure 3J). Taken altogether, these results revealed that the induction of apoptosis by MbovP280 depends on the expression of CRYAB and the amino acids 210–269 in MbovP280 constitute an essential domain for this function.

### CRYAB is a Ligand of MbovP280

**Figure 4:**
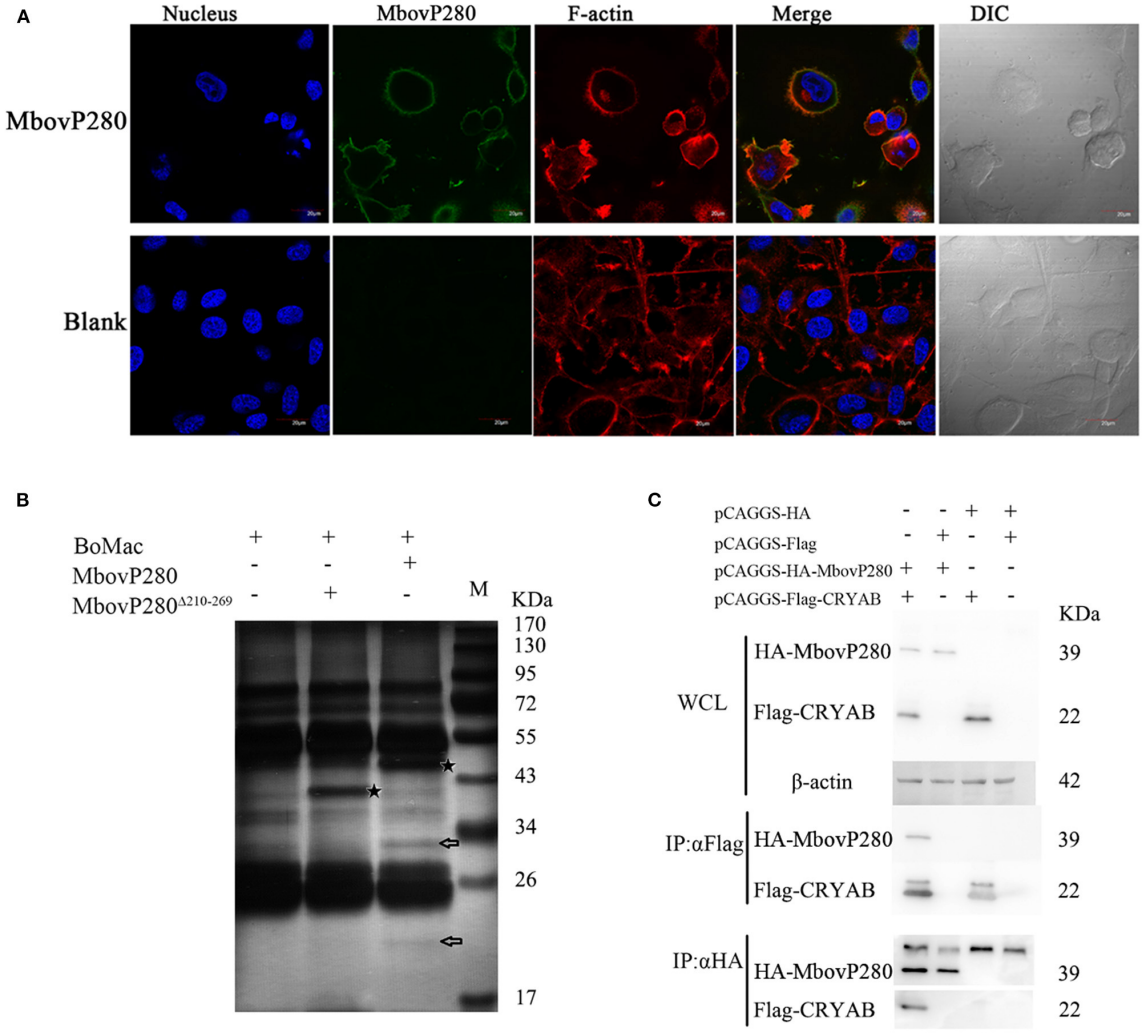
Interaction between MbovP280 and BoMac. **(A)** Binding assay of MbovP280 to BoMac cells. BoMac cells (1 ×10^5^) were incubated with 0.5 μM MbovP280 for 1 h at 37°C. MbovP280 was probed with polyclonal antibody directed against MbovP280 (1:500) and Alexa-Fluor-488-conjugated anti-IgG antibody (1:1,000) (green). Nuclei were stained with DAPI (5 μg/ml) and actin filaments with rhodamine phalloidin (100 nM). **(B)** Identification of MbovP280-binding ligands with mass spectrometry. BoMac cell lysates were incubated with MbovP280 (10 μg), MbovP280^Δ210−269^ (10 μg), or PBS, and immunoprecipitated (IP) with a polyclonal antibody directed against MbovP280 (5 μg) and agarose bead (50 μl) labeled with Protein A/G. Arrows indicate specific MbovP280-binding ligands resolved with SDS-PAGE. And the asterisks represent either MbovP280 or MbovP280^Δ210−269^. **(C)** Interaction between MbovP280 and CRYAB. At 32 h post transfection with plasmids encoding HA, HA–MbovP280, Flag, or Flag–CRYAB, HEK293T cells were harvested and the whole-cell lysates were immunoprecipitated with commercial antibodies directed against either the HA or Flag tag, and Protein A/G agarose beads. MbovP280 and CRYAB were then detected with western blotting assays using the antibodies against either the HA or Flag tags.

Confocal laser microscopy was used to observe the binding of MbovP280 to BoMac. As shown in Figure 4A, rMbovP280 bound strongly to BoMac cells after it was incubated with the cells for 1 h at 37°C, demonstrated by the co-localization (merged yellow signal) of rMbovP280 (green) and F-actin (red).

Further the IP–MS method was to screen for MbovP280-binding proteins by using the antisera against rMbovP280 and rMbovP280^Δ210−269^ to catch the interactive components from lysates of the BoMac cells, separated by Protein A/G Agarose Beads and resolved by SDS-PAGE and specific bands were analyzed with mass spectrometry (MS). As a result, two specific bands (arrows) from BoMac were precipitated by rMbovP280, but not by rMbovP280^Δ210−269^. And the asterisks represent MbovP280 or MbovP280^Δ210−269^ (Figure 4B). Then the bands were subject to be assayed with MS and the resultant proteomics data have been deposited to the ProteomeXchange Consortium via the PRIDE partner repository with the dataset identifier PXD022080. From the MS data, CRYAB, endothelin 2, lipocalin 2, THO complex subunit 4, and DCN1-like protein were preliminarily identified. Among these, CRYAB had the highest percentage coverage (12.57%) (Table 3). CRYAB is a known anti-apoptosis protein, which is consistent with our previous finding that MbovP280 induced the apoptosis of its host cells. Therefore, further experiments were performed to verify the interaction between CRYAB and MbovP280.

**Table 3:** MbovP280-binding ligands in BoMac screened with IP-MS.

**Protein**	**UniProt ID**	**Unique Peptide Count**	**Percentage coverage**	**MW (kDa)**	**PI**
α-Crystallin B chain	V6F832	2	12.57%	20.1	6.76
Endothelin 2	Q867A9	1	2.82%	19.7	10.46
Lipocalin 2	E1B6Z6	1	3.50%	22.9	9.35
THO complex subunit 4	Q3T0I4	2	7.00%	26.9	11.15
DCN1-like protein	F1MRG9	1	1.93%	29.6	6.21

HEK293T cells were cotransfected with plasmids expressing Flag–CRYAB and HA–MbovP280, and a Co-IP assay was performed. The total cellular proteins were prepared and the components immunoprecipitated with either the anti-Flag or anti-HA antibodies. The precipitates were resolved with SDS-PAGE and analyzed with either the anti-HA or anti-Flag antibody by western blotting assays. As shown in Figure 4C, Supplementary Figure 4, the coprecipitated MbovP280 was detected in the compounds precipitated with anti-Flag antibody, and the coprecipitated CRYAB was detected in the compounds precipitated with anti-HA antibody. These results confirmed that MbovP280 specifically interacted with CRYAB.

## Discussion

A number of software are usually used to predict the secreted proteins of *Mycoplasma* species *in silico*. The SignalP4.1 server is used to identify the classically secreted proteins, such as P80 of *M. hominis* (11), which are secreted after the cleavage of the signal peptide, and the SecretomeP 2.0 server is used to predict the non-classical secreted proteins, which may be secreted independently of any signal peptide (27), for example via the extracellular vesicles recently identified in *M. mycoides* subsp. *mycoides* (16) and *Acholeplasma laidlawii* PG8 (28). However, these predictions should be confirmed with other methods, such as western blotting assays. During this study, only two of 11 predicted secreted proteins (MbovP280 and MbovP475) were confirmed as both highly expressed and immunogenic (Figure 1B) suggesting the difficulties in identifying the secreted proteins of mycoplasmal species. Several factors might affect this process, including the reliability of prediction, the relative times and levels of expression and the immunogenicity of the secreted proteins, the complex background of proteins in the media (derived from serum and yeast extracts), and the strong contamination of the secretomes with cytoplasmic proteins during the extraction process (29). Because the lipoproteins of mycoplasmas usually function as virulence factors, such as OppA, a lipoprotein of both *M. agalactiae* and *M. fermentans* that induces apoptosis (16, 30), the secreted lipoproteins MbovP280 and MbovP475 could play critical roles in the pathogenesis of *M. bovis*.

It has previously been reported that *M. bovis* functions as an inducer or inhibitor of the apoptosis of infected cells, probably dependent on the strain or cell type infected (31, 32). For example, *M. bovis* induced the apoptosis of bovine peripheral blood mononuclear cells (PBMCs) (31) and macrophages (33), but is also reported to inhibit the apoptosis of PBMCs, monocytes, primary bovine macrophages, and bovine macrophages (32, 34–36). However, few studies have determined the specific mechanism responsible for this effect.

In this study, we first demonstrated that MbovP280 significantly reduced cell viability and then confirmed it induced the apoptosis of BoMac when applied as either mycoplasmal cells or the purified recombinant protein. However, there is a large discrepancy in proportions of apoptotic cells between induction of purified rMbovP280 (25–80%) and *M. bovis* infection (about 15%). We think this discrepancy might be caused by following reasons. Firstly, the amount of purified rMbovP280 used to treat BoMac is larger than the amount of MbovP280 naturally expressed by the HB0801 strains. Secondly, in *M. bovis* strains, there may be other proteins except MbovP280 function as either inducers or inhibitors of the apoptosis. Therefore, the overall effect of MbovP280 in the strain on apoptosis might be partially contradicted. Since the secreted proteins in the cultural supernatant is very low, the further study should verify whether the concentration of secreted MbovP280 of *M. bovis* increases or not under natural infection and the increase concentration of MbovP280 is necessary to induce apoptosis.

**Figure 5:**
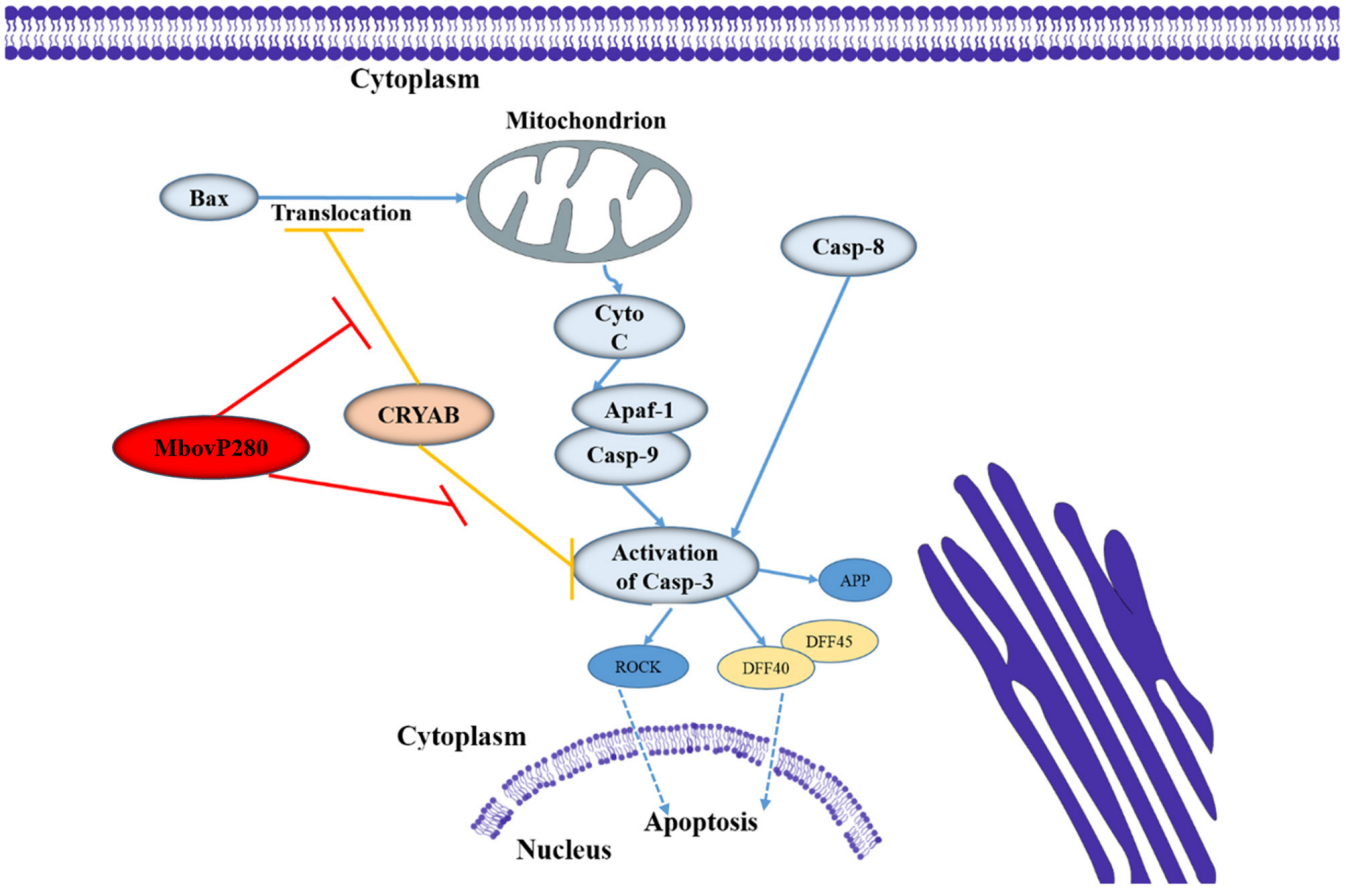
Diagram of the possible signaling pathway of MbovP280-induced apoptosis in BoMac. MbovP280 binds to CRYAB and induces apoptosis of BoMac cells through CRYAB.

CRYAB is a molecular chaperone that is induced by stress and suppresses the aggregation of denatured proteins (37). It belongs to the conserved small heat shock protein family and is highly constitutively expressed in human cancers. Recent studies have shown that CRYAB inhibits apoptosis during myogenic differentiation (38) or differentiation induced by a wide range of stimuli, such as staurosporine, tumor necrosis factor α (TNF-α), UVA irradiation, okadaic acid, hydrogen peroxide, and TNF-related apoptosis-inducing ligand (TRAIL) (39–44). Several studies have demonstrated that CRYAB inhibits apoptosis by suppressing the activation of caspase 3 by interacting with its precursor (38, 45), and by interacting with Bcl-XS and Bax to suppress their translocation (41, 46). In the present study, IP-MS, and Co-IP methods were used to identify the interaction between MbovP280 and CRYAB in BoMac, and confirmed that CRYAB is a specific ligand of MbovP280. A homology model of CRYAB, MbovP280, and caspase 3 was established with SWISS-MODEL, and protein–protein docking was examined with ClusPro 2.0. The results suggested that MbovP280 and the caspase 3 precursor competitively bind to the surface pocket of the CRYAB protein (Supplementary Figure 5) to suppress the anti-apoptosis effect of CRYAB (Figure 5).

In conclusion, in this study, we have demonstrated that MbovP280 is a highly expressed and immunogenic secreted protein that induces the apoptosis of BoMac through its ligand CRYAB and the functional domain is located at amino acids 210–269 which form a coiled-coil domain.

## Data Availability Statement

The datasets presented in this study can be found in online repositories. The names of the repository/repositories and accession number(s) can be found in the article/Supplementary Material.

## Ethics Statement

The animal study was reviewed and approved by Experimental Animal Ethics Committee of Huazhong Agricultural University.

## Author Contributions

GZ, HZ, and AG: study design. GZ and XZ: study conduct. YC, CH, and HC: data analysis and interpretation. GZ, AG, and ES: wrote the manuscript with all authors providing feedback.

## Conflict of Interest

The authors declare that the research was conducted in the absence of any commercial or financial relationships that could be construed as a potential conflict of interest.

## Supplementary Material

Supplementary Figure 1 Secretion of predicted secreted proteins was detected with western blotting assays. Secretome (S) and whole-cell lysate (WCL) of *M. bovis* were resolved with SDS-PAGE, transferred onto the polyvinylidene difluoride membranes, and immunodetected with polyclonal antibodies in the antisera directed against each predicted protein.

Supplementary Figure 2 Verification of secreted MbovP280 and MbovP475 in culture supernatant with western blotting assays. *M. bovis* HB0801 was cultured in PPLO medium and culture supernatant was collected and concentrated at 6, 12, 24, and 36 h. Polyclonal antibodies in the antisera against rMbovP280 and rMbovP475 were used to detect the proteins in the supernatant, while the known *M. bovis* membrane-associated protein NOX served as negative control.

Supplementary Figure 3 MbovP280 increased the levels of cleaved caspase-3. The cell lysates of BoMac cells treated with 0.5 μM rMbovP280 or rMbovP280^Δ210−269^ or infected with *M. bovis* strains (MOI = 1,000) were resolved with SDS-PAGE, transferred onto the polyvinylidene difluoride membrane, and then immunodetected with the antibody directed against cleaved caspase-3. β-actin was used as the internal control.

Supplementary Figure 4 Interaction between MbovP280 and CRYAB. **(A)** Expression of MbovP280 and CRYAB in HEK293T cells at 32 h after transfection with the plasmids encoding HA or HA–MbovP280 together with the plasmids encoding Flag or Flag–CRYAB. **(B)** Interaction between MbovP280 and CRYAB was detected with a western blotting assay.The cell lysates were immunoprecipitated with the antibody against the Flag tag and immunoblotted with the antibody against the HA tag. **(C)** Interaction between MbovP280 and CRYAB was detected with a western blotting assay. The cell lysates were immunoprecipitated with the antibody against the HA tag and immunoblotted with the antibody against the Flag tag.

Supplementary Figure 5 Images of protein–protein docking. **(A)** Homology model of CRYAB and the MbovP280 functional domain (amino acids 206–294) was generated with SWISS-MODEL. Protein–protein docking between CRYAB and MbovP280 (amino acids 206–294) was established with ClusPro 2.0. **(B)** Homology model of CRYAB and caspase 3 was generated with SWISS-MODEL. Protein–protein docking between CRYAB and caspase 3 was established with ClusPro 2.0.

Supplementary Table 1 The predicted secreted lipoproteins of *Mycoplasma bovis*.
