# Supplementary figures and images for: Novel Secreted Protein of Mycoplasma bovis MbovP280 Induces Macrophage Apoptosis Through CRYAB
Source: Front Immunol. 2021 Feb 15;12:619362. doi: 10.3389/fimmu.2021.619362 (PMC7917047; doi:10.3389/fimmu.2021.619362)

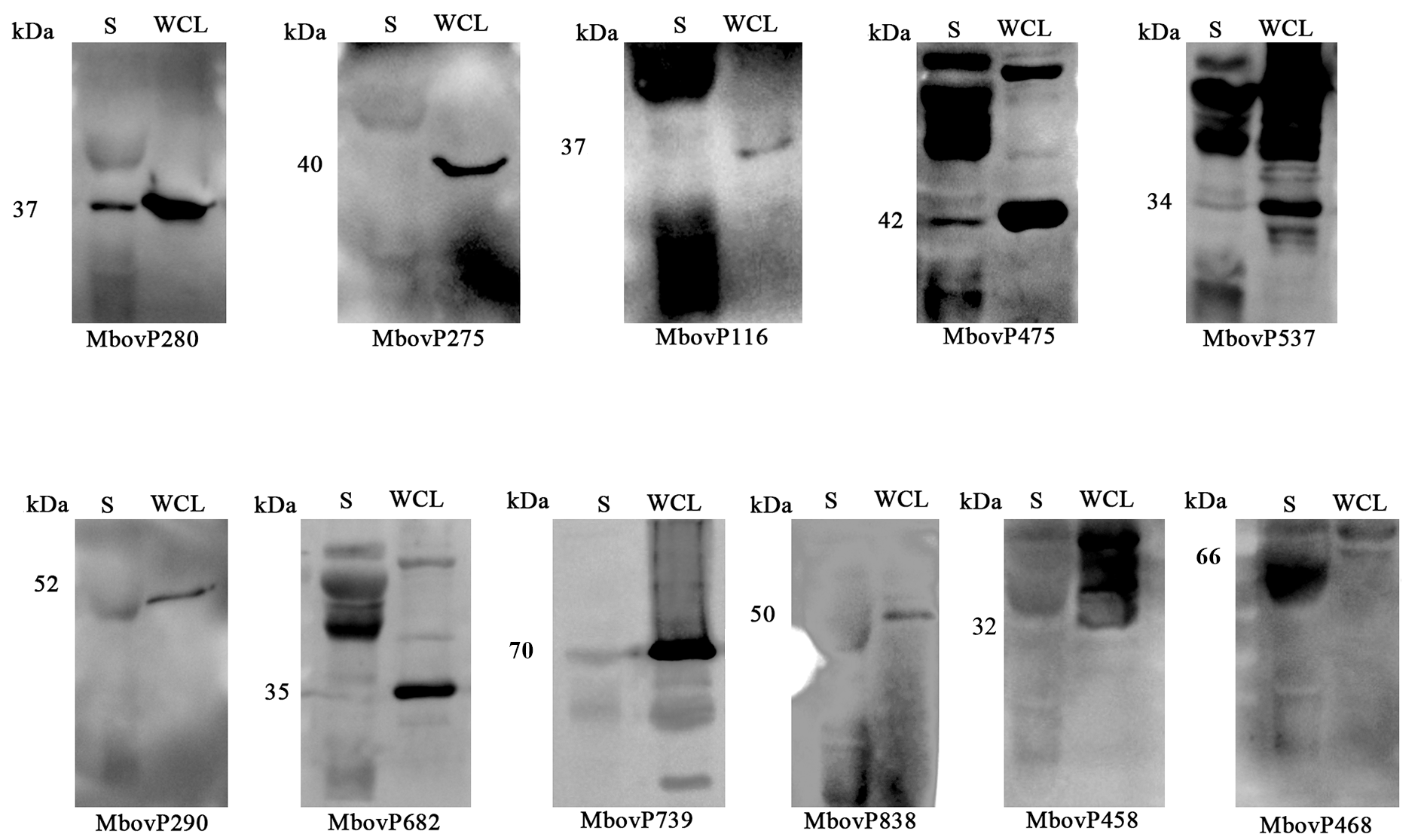

Supplement: Supplementary Figure 1 — Secretion of predicted secreted proteins was detected with western blotting assays. Secretome (S) and whole-cell lysate (WCL) of M. bovis were resolved with SDS-PAGE, transferred onto the polyvinylidene difluoride membranes, and immunodetected with polyclonal antibodies in the antisera directed against each predicted protein. [file Image_1.TIF]

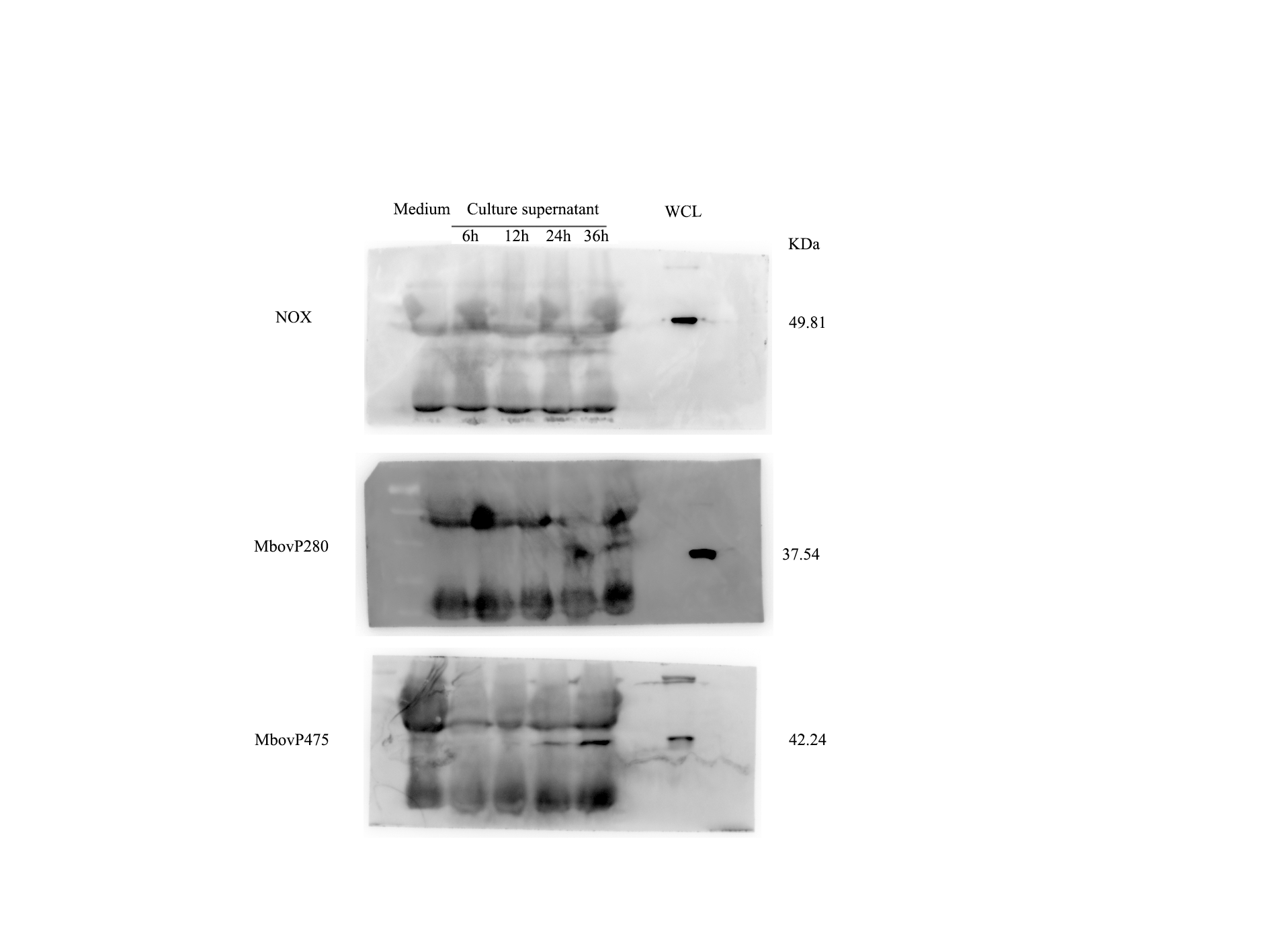

Supplement: Supplementary Figure 2 — Verification of secreted MbovP280 and MbovP475 in culture supernatant with western blotting assays. M. bovis HB0801 was cultured in PPLO medium and culture supernatant was collected and concentrated at 6, 12, 24, and 36 h. Polyclonal antibodies in the antisera against rMbovP280 and rMbovP475 were used to detect the proteins in the supernatant, while the known M. bovis membrane-associated protein NOX served as negative control. [file Image_2.TIF]

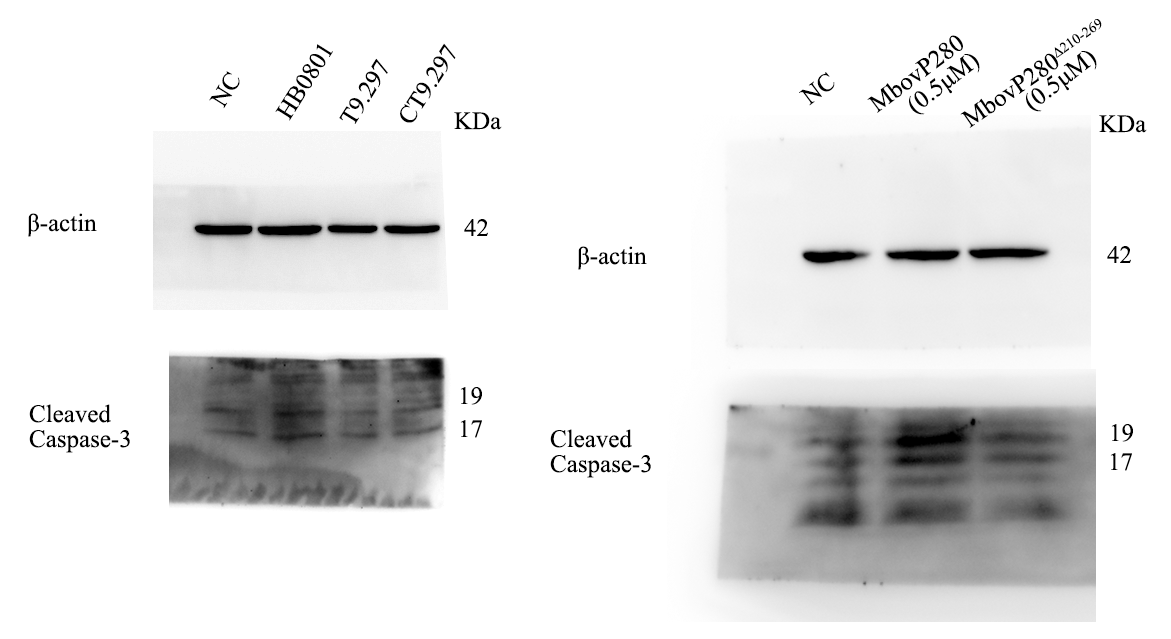

Supplement: Supplementary Figure 3 — MbovP280 increased the levels of cleaved caspase-3. The cell lysates of BoMac cells treated with 0.5 μM rMbovP280 or rMbovP280Δ210−269 or infected with M. bovis strains (MOI = 1,000) were resolved with SDS-PAGE, transferred onto the polyvinylidene difluoride membrane, and then immunodetected with the antibody directed against cleaved caspase-3. β-actin was used as the internal control. [file Image_3.TIF]

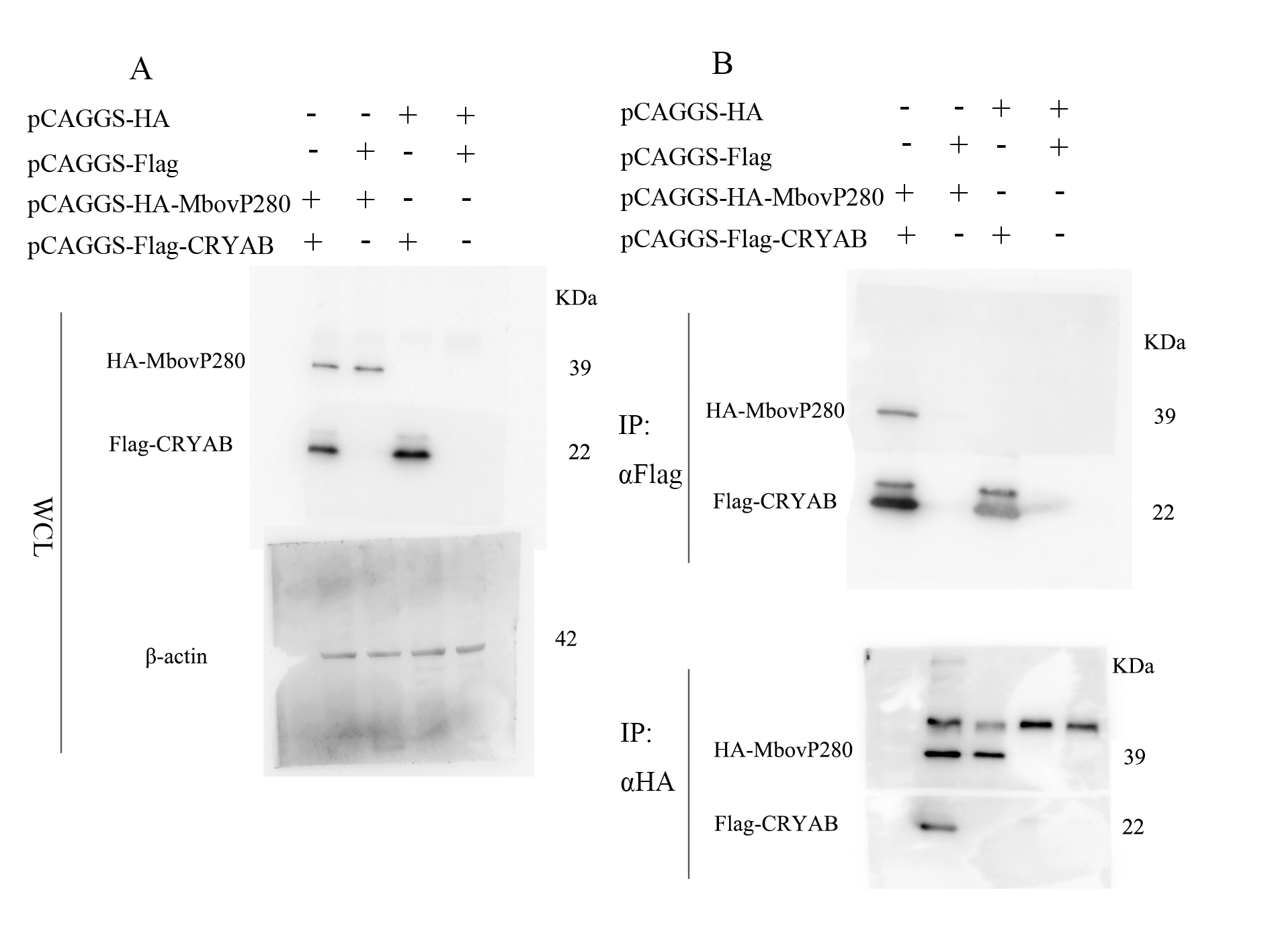

Supplement: Supplementary Figure 4 — Interaction between MbovP280 and CRYAB. (A) Expression of MbovP280 and CRYAB in HEK293T cells at 32 h after transfection with the plasmids encoding HA or HA–MbovP280 together with the plasmids encoding Flag or Flag–CRYAB. (B) Interaction between MbovP280 and CRYAB was detected with a western blotting assay.The cell lysates were immunoprecipitated with the antibody against the Flag tag and immunoblotted with the antibody against the HA tag. (C) Interaction between MbovP280 and CRYAB was detected with a western blotting assay. The cell lysates were immunoprecipitated with the antibody against the HA tag and immunoblotted with the antibody against the Flag tag. [file Image_4.TIF]

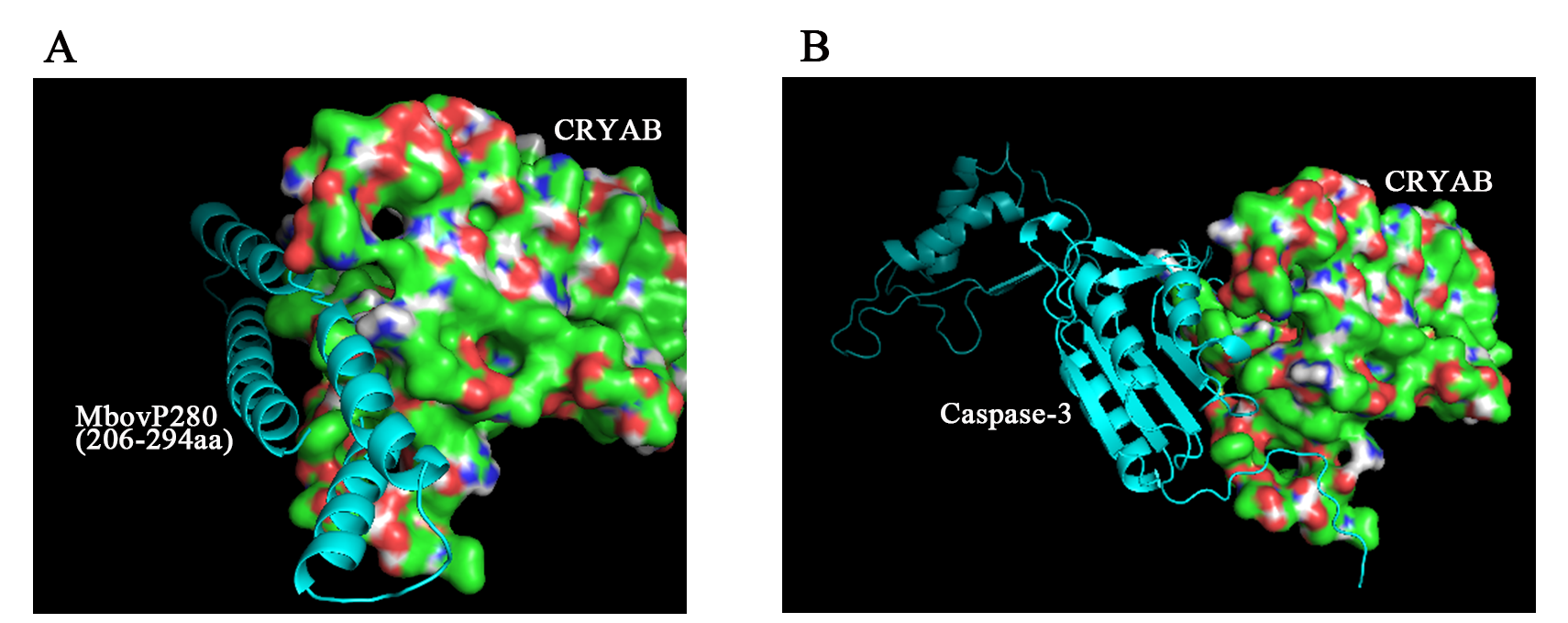

Supplement: Supplementary Figure 5 — Images of protein–protein docking. (A) Homology model of CRYAB and the MbovP280 functional domain (amino acids 206–294) was generated with SWISS-MODEL. Protein–protein docking between CRYAB and MbovP280 (amino acids 206–294) was established with ClusPro 2.0. (B) Homology model of CRYAB and caspase 3 was generated with SWISS-MODEL. Protein–protein docking between CRYAB and caspase 3 was established with ClusPro 2.0. [file Image_5.TIF]
