# Supplementary material for: Novel Secreted Protein of Mycoplasma bovis MbovP280 Induces Macrophage Apoptosis Through CRYAB
Source: Front Immunol. 2021 Feb 15;12:619362. doi: 10.3389/fimmu.2021.619362 (PMC7917047; doi:10.3389/fimmu.2021.619362)
Supplement: Supplementary Table 1 — The predicted secreted lipoproteins of Mycoplasma bovis. [file Table_1.DOCX]

| **Name** | **ORF** | **SignalP-TM Score Cl** | **SecP Score NC** |
| --- | --- | --- | --- |
| lipoprotein | Mbov_0393 | 0.465 | 0.894972 |
| lipoprotein | Mbov_0739 | 0.466 | 0.83488 |
| lipoprotein | Mbov_0477 | 0.483 | 0.825501 |
| lipoprotein | Mbov_0374 | 0.5 | 0.896303 |
| lipoprotein | Mbov_0537 | 0.502 | 0.81691 |
| lipoprotein | Mbov_0538 | 0.506 | 0.861251 |
| lipoprotein | Mbov_0274 | 0.53 | 0.824769 |
| lipoprotein | Mbov_0570 | 0.532 | 0.83684 |
| lipoprotein | Mbov_0119 | 0.537 | 0.838733 |
| lipoprotein | Mbov_0275 | 0.55 | 0.906692 |
| lipoprotein | Mbov_0465 | 0.55 | 0.868609 |
| P48-like lipoprotein | Mbov_0016 | 0.559 | 0.812986 |
| lipoprotein | Mbov_0546 | 0.56 | 0.836718 |
| lipoprotein | Mbov_0350 | 0.566 | 0.921368 |
| lipoprotein | Mbov_0289 | 0.576 | 0.888045 |
| lipoprotein | Mbov_0475 | 0.579 | 0.83653 |
| lipoprotein | Mbov_0585 | 0.583 | 0.825032 |
| lipoprotein | Mbov_0592 | 0.583 | 0.833952 |
| lipoprotein | Mbov_0217 | 0.587 | 0.854821 |
| lipoprotein | Mbov_0280 | 0.602 | 0.766504 |
| lipoprotein | Mbov_0659 | 0.609 | 0.819394 |
| lipoprotein | Mbov_0116 | 0.61 | 0.867306 |
| lipoprotein | Mbov_0339 | 0.613 | 0.932253 |
| lipoprotein | Mbov_0458 | 0.615 | 0.934772 |
| lipoprotein | Mbov_0468 | 0.617 | 0.907307 |
| lipoprotein | Mbov_0654 | 0.618 | 0.936725 |
| lipoprotein | Mbov_0290 | 0.619 | 0.772005 |
| lipoprotein | Mbov_0084 | 0.622 | 0.839226 |
| lipoprotein | Mbov_0347 | 0.623 | 0.894432 |
| lipoprotein | Mbov_0536 | 0.635 | 0.645918 |
| lipoprotein | Mbov_0447 | 0.652 | 0.931656 |
| Vpma-like, lipoprotein | Mbov_0292 | 0.665 | 0.766334 |
| lipoprotein | Mbov_0469 | 0.666 | 0.957223 |
| lipoprotein | Mbov_0838 | 0.691 | 0.91944 |
| lipoprotein | Mbov_0525 | 0.693 | 0.981177 |
| lipoprotein | Mbov_0462 | 0.696 | 0.931796 |
| lipoprotein | Mbov_0461 | 0.708 | 0.873049 |
| lipoprotein | Mbov_0473 | 0.709 | 0.925081 |
| lipoprotein | Mbov_0682 | 0.722 | 0.904195 |

**Table S1** The predicted secreted lipoproteins of *Mycoplasma bovis*
